# Mechanism for transmission and pathogenesis of carbapenem-resistant Enterobacterales harboring the carbapenemase IMP and clinical countermeasures

**DOI:** 10.1128/spectrum.02318-23

**Published:** 2024-01-10

**Authors:** Chengru Yang, Shan Jiang, Chunli Wei, Chunjiang Li, Jianmin Wang, Xinhui Li, Lingyi Zeng, Kewang Hu, Yang Yang, Jisheng Zhang, Xiaoli Zhang

**Affiliations:** 1 Department of Microbiology, Yongchuan Hospital of Chongqing Medical University, Chongqing, China; 2 Department of Microbiology, The First Affiliated Hospital of Jiamusi University, Jiamusi, China; 3 Department of Microbiology, Jiangyou People’s Hospital, Jiangyou, China; 4 Department of Life Science and Technology, Mudanjiang Normal University, Mudanjiang, China; 5 Department of Microbiology, Hangzhou Center for Disease Control and Prevention, Hangzhou, China; 6 Department of Microbiology, Affiliated Hangzhou Xixi Hospital, Zhejiang University School of Medicine, Hangzhou, China; University of Southern California, Duarte, California, USA

**Keywords:** carbapenem-resistant Enterobacterales, IMP, *traD*, imipenem, tigecycline, bactericidal effect

## Abstract

Carbapenem-resistant Enterobacterales (CRE) are some of the most important pathogens causing infections, which can be challenging to treat. We identified four *bla*_IMP_-carrying CRE isolates and collected clinical data. The transferability and stability of the plasmid were verified by conjugation, successive passaging, and plasmid elimination assays. The IncC *bla*_IMP-4_-carrying pIMP4-ECL42 plasmid was successfully transferred into the recipient strain, and the high expression of *traD* may have facilitated the conjugation transfer of the plasmid. Interestingly, the plasmid showed strong stability in clinical isolates. Whole-genome sequencing was performed on all isolates. We assessed the sequence similarity of *bla*_IMP_ -harboring plasmid from our institution and compared it to plasmids for which sequence data are publicly available. We found that four *bla*_IMP_-carrying CRE belonged to four different sequence types. The checkerboard technique and time-kill assays were used to investigate the best antimicrobial therapies for *bla*_IMP_-carrying CRE. The time-kill assay showed that the imipenem of 1× minimum inhibitory concentration (MIC) alone had the bactericidal or bacteriostatic effect against IMP-producing strains at 4–12 h *in vitro*. Moreover, the combination of tigecycline (0.5/1/2 × MIC) and imipenem (0.5/1 × MIC) showed a bactericidal effect against the *bla*_IMP-26_-carrying CRECL60 strain.

**IMPORTANCE:**

Carbapenem-resistant Enterobacterales (CRE) are an urgent public health threat, and infections caused by these microorganisms are often associated with high mortality and limited treatment options. This study aimed to determine the clinical features, molecular characteristics, and plasmid transmissible mechanisms of *bla*_IMP_ carriage as well as to provide a potential treatment option. Here, we demonstrated that conjugated transfer of the IncC *bla*_IMP-4_-carrying plasmid promotes plasmid stability, so inhibition of conjugated transfer and enhanced plasmid loss may be potential ways to suppress the persistence of this plasmid. The imipenem alone or tigecycline-imipenem combination showed a good bactericidal effect against IMP-producing strains. In particular, our study revealed that imipenem alone or tigecycline-imipenem combination may be a potential therapeutic option for patients who are infected with IMP-producing strains. Our study supports further trials of appropriate antibiotics to determine optimal treatment and emphasizes the importance of continued monitoring of IMP-producing strains in the future.

## INTRODUCTION

Enterobacterales are common causes of community-acquired and hospital-acquired infections (1). Carbapenems are often considered the most effective antibacterial drugs for the treatment of serious infections with many resistant Enterobacterales. However, with the widespread clinical use of carbapenems, carbapenem-resistant Enterobacterales (CRE) have emerged in a wide range in the world and have become the focus of the global antimicrobial community (2, 3). The main mechanism of resistance to carbapenems in Enterobacterales is the production of carbapenemases (2). The IMP-type metallo-β-lactamase (MBL) is one of the most common families of acquired carbapenemases detected in Enterobacterales and is mobilized within the insertion sequence *IS26* (4, 5). Among them, mobile MBL (IMP-1) was the first identified cause of acquired resistance to carbapenems in Gram-negative bacilli (6). The *bla*_IMP-4_ was initially detected in isolates of *Acinetobacter* spp. obtained from blood and wounds of humans in Hong Kong from 1994 to 1998 (7). However, *bla*_IMP-26_ is a variant of *bla*_IMP-4_ that was originally identified in a clinical isolate of *Pseudomonas aeruginosa* isolated in Singapore in 2008 (8).

Almost all *bla*_IMP_ are located on plasmids (9–11), which are transferred horizontally between Enterobacterales and thus lead to widespread dissemination of antibiotic resistance genes (ARGs), and the main mechanism of this horizontal gene transfer is conjugation (12). The type IV secretion system (T4SS) can mediate bacterial conjugation transfer and is classified into three subtypes: subtypes IVA (F-type encoded by the *tra* gene and P-type encoded by the *trb* gene), IVB (I-type encoded by the Dot/Icm), and GI (all other T4SS with low or no homology with subtypes IVA and IVB) (13, 14). Notably, the vast majority of conjugative plasmids and integrative and conjugative elements in Gram-negative bacteria rely on their own encoded T4SS for DNA and protein transfer, which leads to the spread of antibiotic resistance and pathogenicity (13). As CRE poses a global threat, more antimicrobial therapies are being further considered.

In the present study, we investigated the ARGs, virulence genes, and conjugative transfer-related genes of four CRE strains using PCR, whole-genome sequencing (WGS), and bioinformatic analysis. We determined the transferability and stability of the *bla*_IMP_-harboring plasmids using conjugation, plasmid stability, and plasmid elimination assays. We analyzed WGS data and compared them with publicly available data to determine the genome structure of the *bla*_IMP_-carrying strain. We determined the *in vitro* virulence of strains using biofilm formation and serum complement-mediated killing assays. Moreover, synergistic activity and *in vitro* antibacterial activity of different combinations of antibiotics were determined using checkerboard and time-kill assays.

## RESULTS

### Characteristics of IMP-producing strains

**Fig 1:**
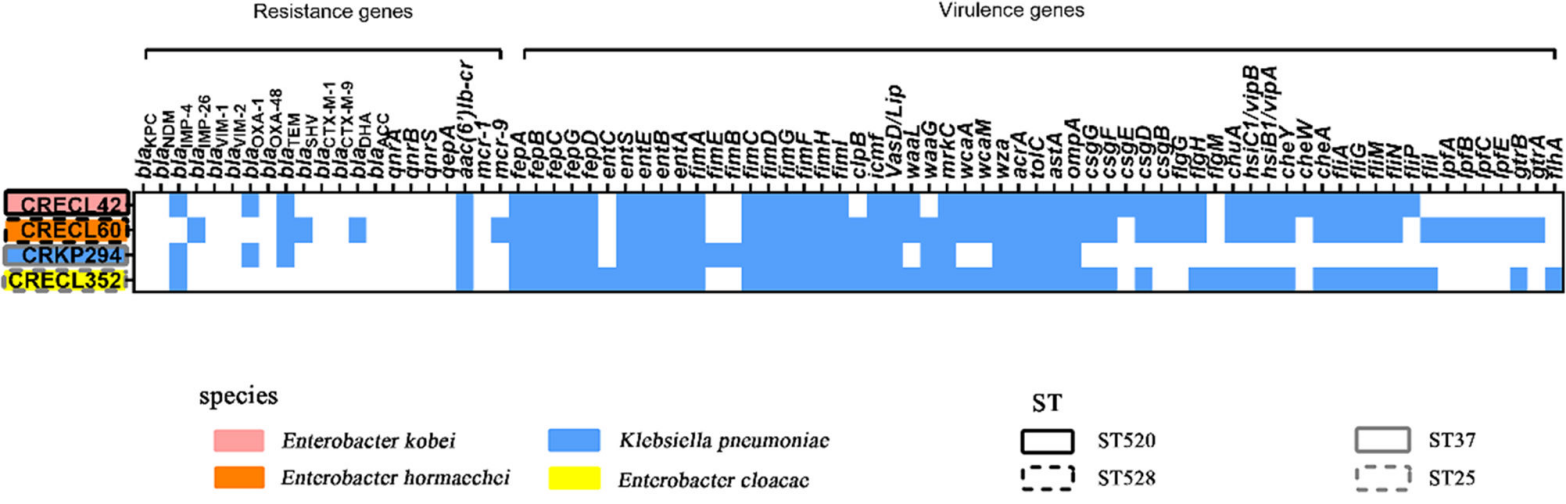
Characteristics of the four IMP-CRE strains collected in this study. Four isolates are shown in different colors. The ST of each strain is shown with a solid or dashed line of a different color. The absence (in white) or presence (in blue) of resistance and virulence genes is displayed by a heatmap.

We collected a total of four non-duplicate CRE strains, including three carbapenem-resistant *Enterobacter cloacae* complex (CRECL) strains and one carbapenem-resistant *Klebsiella pneumoniae* (CRKP) strain. The three CRECL strains belonged to three species: *Enterobacter kobei* (CRECL42, IMP-4-producing), *Enterobacter hormaechei* (CRECL60, IMP-26-producing), and *Enterobacter cloacae* (CRECL352, IMP-4-producing) (Fig. 1). The traceable metadata showed that the strains come from different specimens, including blood (2/4, 50%), sputum (1/4, 25%), and secretion of wound infection after trauma to the left Achilles tendon (1/4, 25%) (Table S2). According to the multi-locus sequence typing (MLST) analysis, four strains were of different ST types ST520 (CRECL42), ST528 (CRECL60), ST37 (CRKP294), and ST25 (CRECL352) (Fig. 1). In addition to *bla*_IMP_, these four CRE strains also carried the β-lactamase genes (*bla*_OXA-1,_*bla*_TEM-1,_*bla*_SHV-12_, or *bla*_DHA-1_) and the quinolone resistance gene (*aac(6')Ib-cr*) (Fig. 1). Notably, we detected the *mcr-9* gene in the CRECL60 strain, but the MIC of polymyxin B (PB) in the CRECL60 strain was 2 mg/L (Fig. 1; Table S2).

### *bla*_IMP-4_ was located on an IncC self-transmissible plasmid

**Fig 2:**
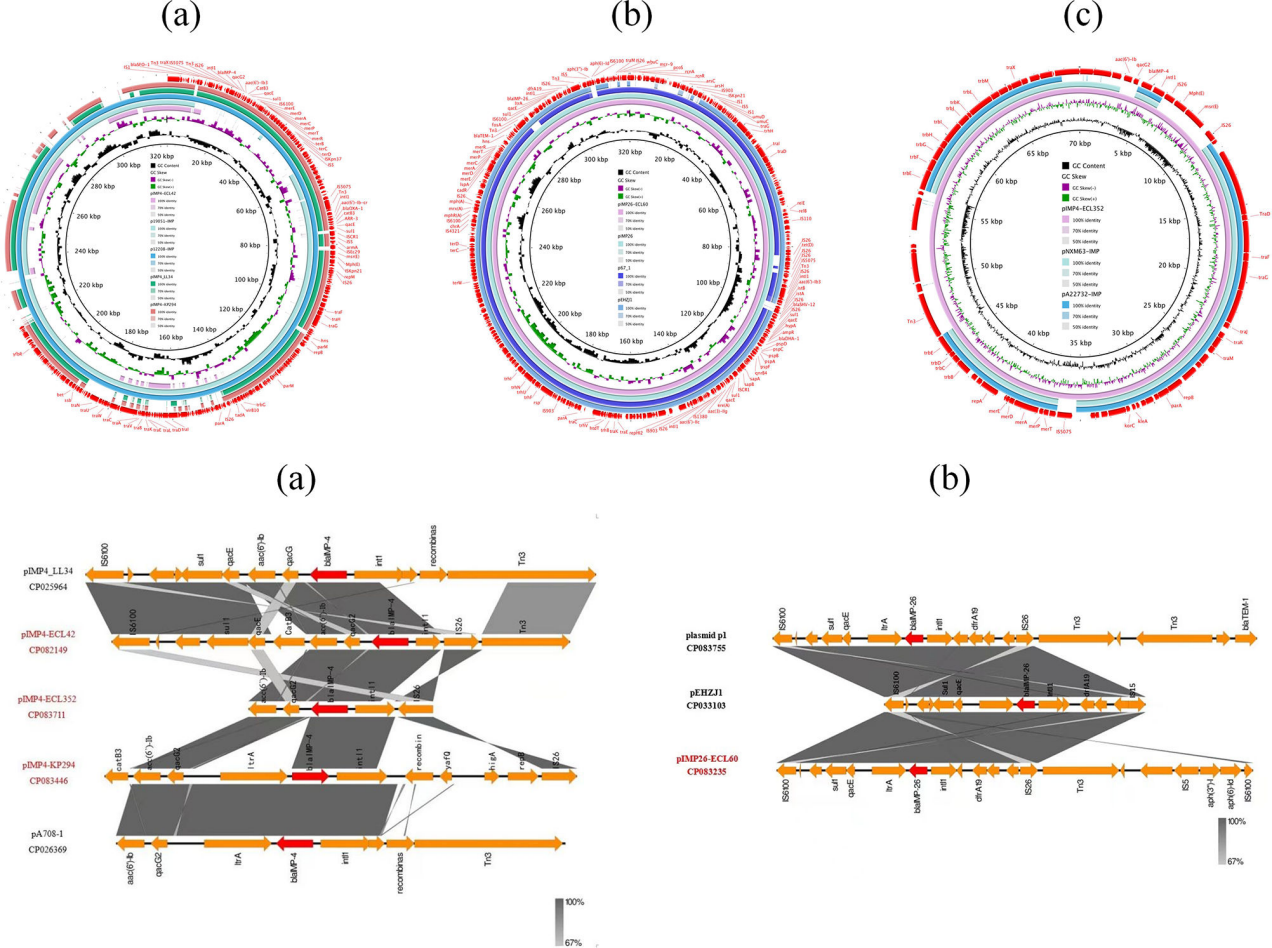
(Top) Sequence comparison of blaIMP-carrying plasmids pIMP4-ECL42, p19051-IMP, p12208-IMP, pIMP4_LL34, and pIMP4-KP294 (**a**); pIMP26-ECL60, pIMP26, p67_1, and pEHZJ1 (**b**); pIMP4-ECL352, pNXM63-IMP, and pA22732-IMP (**c**). The complete plasmid pIMP4-ECL42, pIMP4-KP294, pIMP26-ECL60, and pIMP4-ECL352 sequence was used as the reference, and the white and colored regions of the circles indicate absence and presence, respectively. (Bottom) Different genetic structures surrounding the *bla*_IMP_. Comparison of *bla*_IMP_ regions detected in pIMP4_LL34, pIMP4-ECL42, pIMP4-ECL352, pIMP4-KP294, and pA708-1 (**a**); plasmid p1, pEHZJ1, and pIMP26-ECL60 (**b**). Gray shading denotes regions of shared homology. Arrows indicate the direction of gene transcription.

In our setting, *bla*_IMP-4_- or *bla*_IMP-26_-carrying plasmids belonged to different replicon types (IncC, IncHI2, IncU, or IncP1), and four plasmids were named pIMP4-ECL42, pIMP26-ECL60, pIMP4-KP294, and pIMP4-ECL352, with sizes of 217,402, 320,374, 320,374, and 70,058 bp, respectively (Table S3). pIMP4-ECL42 shared 99.99% identity and 65% coverage with plasmid p19051-IMP (accession no. MF344565.1) carried by *K. pneumoniae* in Beijing, which was identified in 2017 (Fig. 2, top); pIMP26-ECL60 shared 99.99% identity and 95% coverage with plasmid pIMP26 (Fig. 2, top), and the phylogenetic tree also showed a link between pIMP26-ECL60 and pIMP26 (accession no. MH399264), which was isolated from an *E. cloacae* isolate from Shanghai, China (Fig. S1A); pIMP4-KP294 shared 99.96% identity and 71% coverage with plasmid pIMP4_LL34 (accession no. CP025964.2) carried by *K. pneumoniae* in Chengdu, Sichuan, which was identified in 2017 (Fig. 2, top); pIMP4-ECL352 shared 99.99% identity and 75% coverage with plasmid pNXM63-IMP (accession no. MW150990.1) carried by *Morganella morganii* in Guangzhou, Guangdong, which was identified in 2020 (Fig. 2, top). In addition, the phylogenetic tree showed that pIMP4-ECL352 clustered with *bla*_IMP-4_-carrying plasmids from *K. pneumoniae* isolated from Australia (Fig. S1B). Our comparison of four plasmids from our institution revealed that these four plasmids carried multiple ARGs in addition to *bla*_IMP_. These included *bla*_DHA-1_, *bla*_SHV-12_, *bla*_SFO-1_, *bla*_OXA-1_, *bla*_PER-1_, and *bla*_TEM-1B_ for β-lactamase resistance; *aac(6')-lb3*, *armAC*, *aph(3'')-lb*, *aph(6)-ld*, *aadA5*, *aac(6')-llc*, and *aac(3)-IId* for aminoglycoside resistance; *dfrA19* and *dfrA1* for trimethoprim resistance; *sul1* for sulfonamide resistance; *tet(D*) and *tet(A*) for tetracycline resistance; *qnrS1*, *qnrB4*, and *aac(6’)-Ib-c*r for quinolone resistance; *msr(E*), *mph(E*), *ere(A*), and *mph(A*) for macrolide resistance; *mcr-9* for colistin resistance; and *ARR-3* for rifampicin resistance (Table S3).

**Fig 3:**
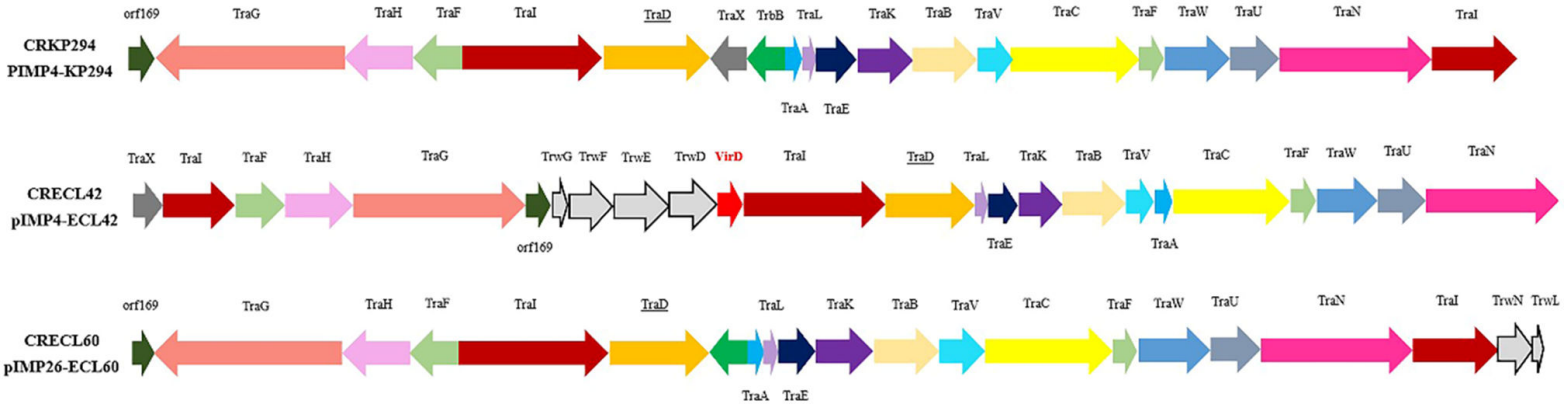
Genetic organization of T4SS (F-type) of CRECL42, CRECL60, and CRKP294. Genes are plotted as arrows in order according to their genomic positions. The underlined text represents the gene whose expression was quantified by RT-qPCR. Different genes are represented by different colors (*virD* is shown in red).

**Fig 4:**
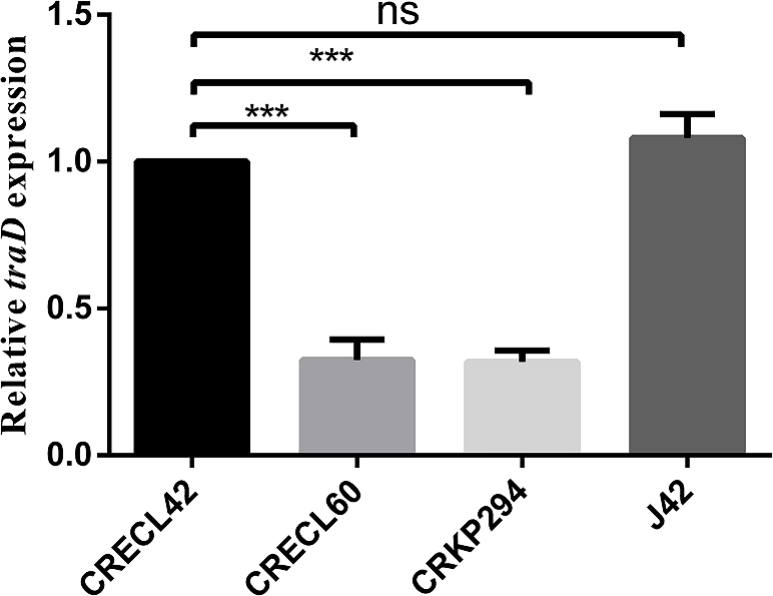
Expression of the *traD* gene in the CRECL42, CRECL60, CRKP294, and J42 strains. Fold changes in mRNA levels were determined by RT-qPCR. *rpoB* was used as a reference gene for normalization. Data represent the mean of three independent experiments performed in triplicate. The asterisks indicate statistical significance at different levels by student’s *t*-tests: ns, not significant; ^***^, *P* ≤ 0.001.

We performed conjugation assays using CRE strains carrying the *bla*_IMP_ as the donor and *Escherichia coli* 600 (rifampicin-resistant) as the recipient strain. There was only one strain of *E. coli* (J42) identified as transconjugant using VITEK-2, 16S rRNA, and PCR (contained *bla*_IMP-4_,*aac(6')-lb*, and *bla*_OXA-1_). The *bla*_IMP-4_-carrying plasmids of the CRECL42 strain could be transferred into recipient *E. coli* 600, at a frequency of 3.9 × 10^−6^ transconjugants per donor cell. Antimicrobial susceptibility testing showed that the J42 strain exhibited resistant phenotypes similar to those of the CRECL42, although the MICs of some agents were lower than those of the donor (Table S2). We also found that the *bla*_IMP-4_-carrying J42 strain has reduced susceptibility to MEM compared to the recipient strain, suggesting that the *bla*_IMP-4_-carrying plasmid accounts for the carbapenem resistance phenotype of J42. We analyzed the conjugative regions of pIMP4-ECL42 and found that it contained four complete modules of self-transmissible plasmids, namely, the transfer origin region (*oriT*), the relaxase gene (*traI*), the gene encoding the type IV coupling protein (T4CP), and the gene cluster for the bacterial T4SS (*traABCEFGHKLNUVW*), indicating that it can be self-transmissible and is consistent with the results of conjugation experiments. We further analyzed the T4SS-mediating bacterial conjugation transfer and found that the four strains were divided into two types: F- (CRECL42, CRECL60, and CRKP294) (Fig. 3) and P-type (CRECL352). Notably, compared to the CRECL42 strain, the relative expression of the *traD* gene was significantly declined in the CRECL60 and CRKP294 strains; however, there was no statistically significant difference in the J42 strain (Fig. 4). Moreover, a comparison of the *bla*_IMP-4_ genetic environment of pIMP4-ECL42, pIMP-4_LL34, and pIMP4-ECL352 revealed a similar structure as *acc(6’)-lb-qacG2-bla*_IMP-4_-*intl1*; pIMP4-KP294 and pA708-1(accession no. CP026369) had the similar structure as *acc(6')-lb-qacG2-ltrA-bla*_IMP-4_*-intl1*. The comparison of the *bla*_IMP-26_ genetic environment of pIMP-26-ECL60, plasmid p1 (accession no. CP083755), and pEHZJ1 (accession no. CP033103) revealed a similar structure as *IS6100-sul1-qacE-ltrA-bla*_IMP-26_*-intl1-drfA19* (Fig. 2, bottom).

### Conjugal transfer contributed significantly to *bla*_IMP-4_-carrying plasmid stability within CRE strains

Plasmid stability analysis showed that the IncP1 pIMP-ECL352 plasmid showed no plasmid loss after 50 consecutive generations of passages in the absence of antibiotics stress; the plasmid of the J42 strain also showed high stability; however, the plasmid retention rates of the IncC pIMP4-ECL42 plasmid and the IncHI2 pIMP26-ECL60 plasmid were only 15% and 8.8%, respectively; notably, the CRKP294 strain lost all plasmids (the IncU pIMP4-KP294 plasmid) after 30 consecutive generations of passages (Tables S3 and S4). For SDS treatment, no plasmids were lost from the CRECL352 and the J42 strains; in contrast, all plasmids were lost from the CRKP294 strain; the plasmid elimination rates were 11.7 and 10% for the CRECL42 and the CRECL60 strains, respectively (Tables S3 and S4).

**Fig 5:**
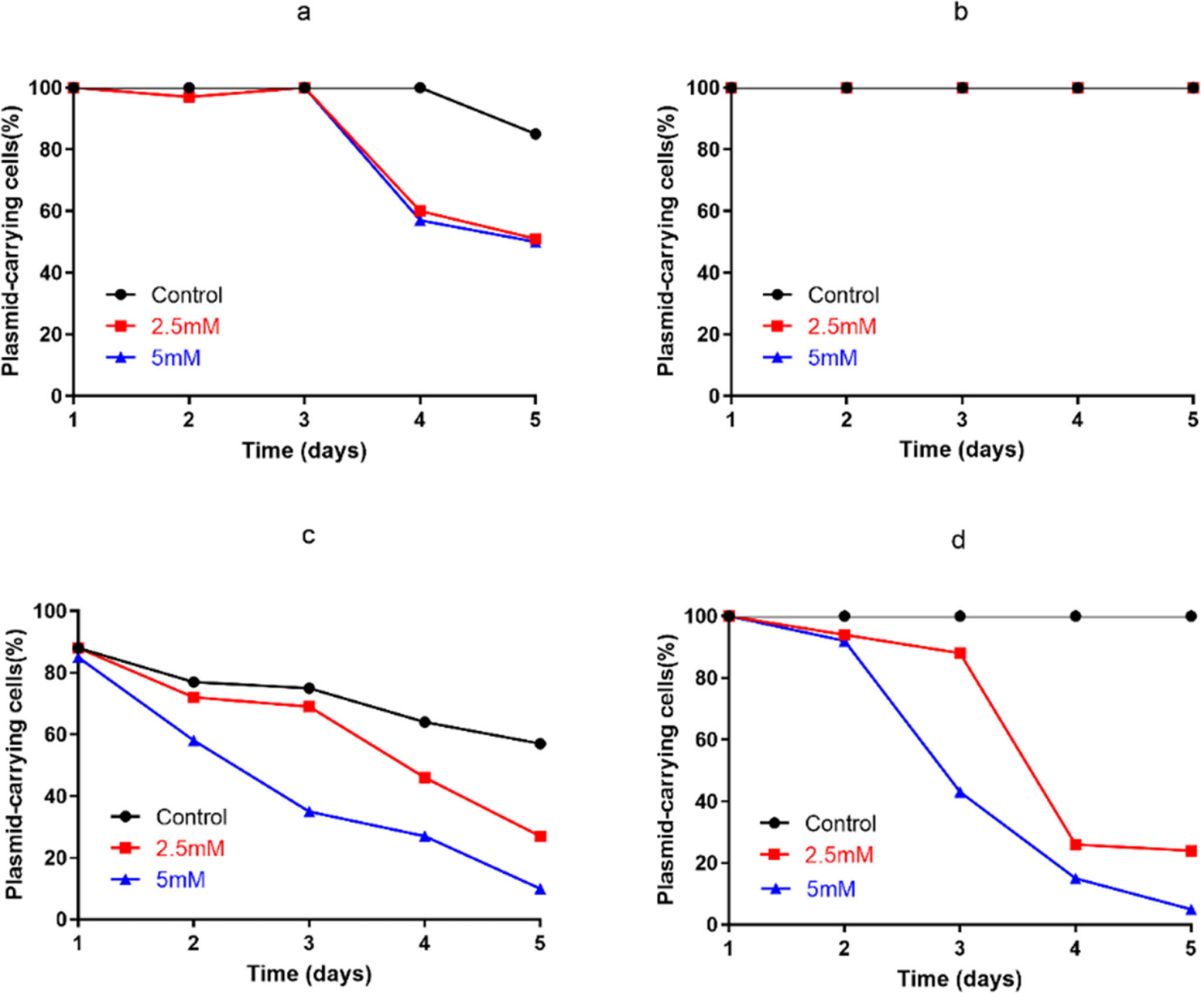
*bla*_IMP_-carrying plasmid stability in strains (a) CRECL42, (b) CRECL60, (c) CRKP294, and (d) CRECL352 cultured with or without linoleic acid.

The IncC *bla*_IMP-4_- (Fig. 5a), IncHI2 *bla*_IMP-26_- (Fig. 5b), and IncP1 *bla*_IMP-4_-carrying plasmids (Fig. 5d) showed strong stability in clinical strains, without apparent plasmid loss after serial subculture for 5 days; however, the IncU *bla*_IMP-4_-carrying plasmid showed significant plasmid loss (Fig. 5c). We also found that after the conjugation inhibitor linoleic acid was added, a gradual increase in the level of *bla*_IMP-4_-carrying plasmid loss could be observed in three strains (CRECL42, CRECL352, and CRKP294); however, the IncHI2 *bla*_IMP-26_-carrying plasmid was not loss (Fig. 5). Moreover, we found that the addition of the conjugation inhibitor linoleic acid reduced the conjugation frequency of the pIMP4-ECL42 plasmid (Fig. S2).

### Biofilm formation, serum resistance, and virulence genes

Compared to the ATCC1705 strain (no IMP-producing), the CRKP294 strain was a stronger biofilm producer, which did not exhibit the capsule serotype and the hypermucoviscous phenotype; however, the CRECL42, CRECL60, and CRECL352 strains were lower (Fig. S3). Therefore, there may be no correlation between *bla*_IMP-4_-harboring strain and biofilm production intensity. The results of the serum complement-mediated killing assay are shown in Fig. S4. The CRKP294 strain showed serum resistance because of a significant growth trend over time (grade 6, 402%); the CRECL42 and CRECL60 strains showed moderate serum sensitivity (grades 3 and 4, 2.4% and 11.6%); however, the CRECL352 strain, which was highly sensitive to serum complement-mediated killing, died within 2 h (grade 1, 0%). Moreover, the virulence gene results showed that all four strains contained type I and III fimbriae, T6SS-encoded, and *csgd* genes (curli gene; except for the CRKP294 strain) etc., but all strains did not contain *papC* and *papD* (*P* pili genes) (Fig. 1).

### Checkerboard synergy analysis

To address which antibiotic combinations might be most clinically useful, we used checkerboard assays. We evaluated 12 combinations of antibiotics and the results are shown in Table S2. There was a synergistic effect of the combination approach of meropenem/levofloxacin (MEM/LEV) against the CRECL42 strain and the combination approach of meropenem/amikacin (MEM/AMK), AMK/LEV, and PB/imipenem (IPM) against the CRECL60 strain (FICI: 0.25 or 0.5). No antagonistic effects were found in any of the antibiotic combination methods. Notably, the results of the tigecycline (TIG)-based checkerboard combination showed no synergistic effect.

### Bacterial time-kill effect

**Fig 6:**
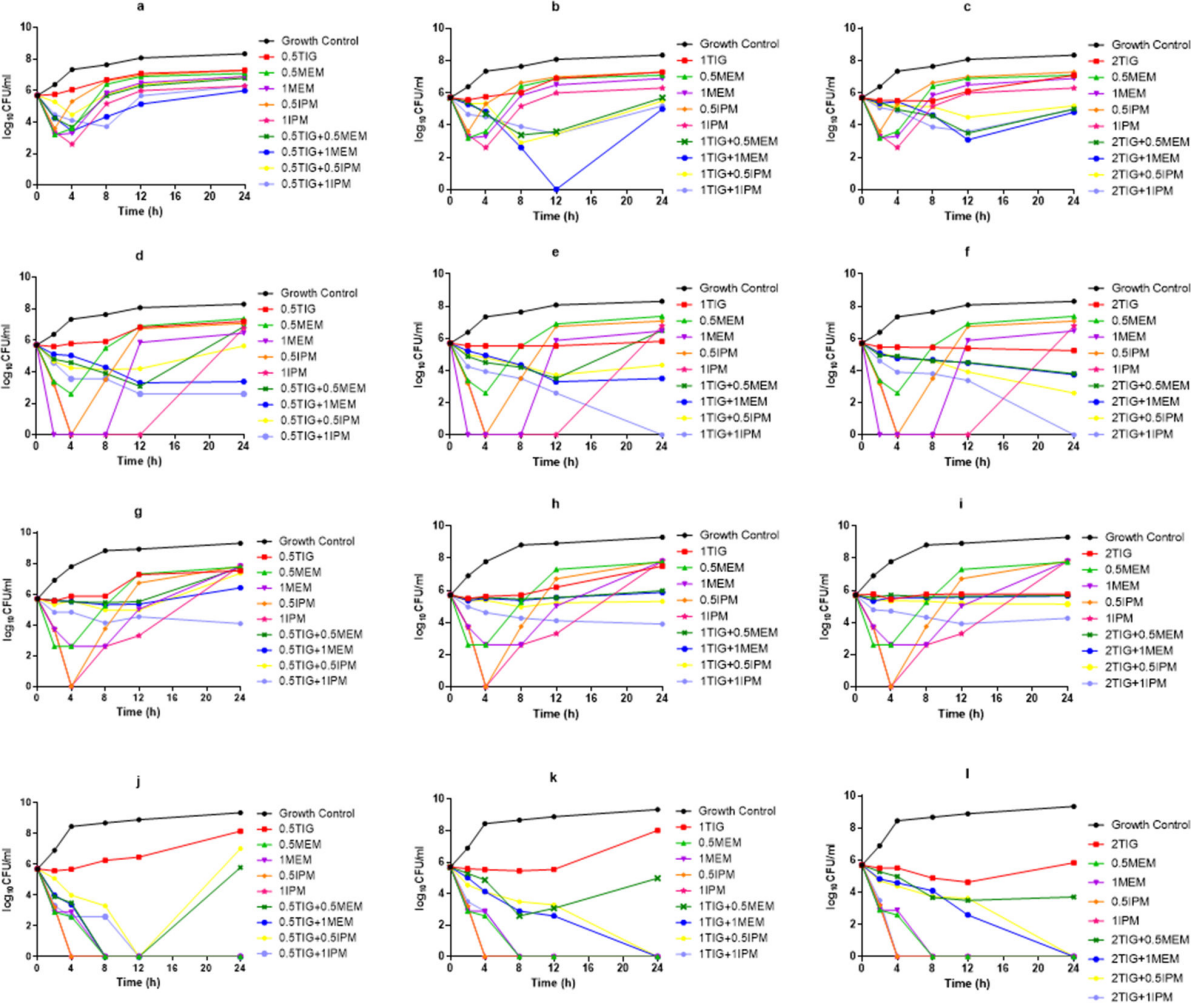
*In vitro* time-kill assays using different concentrations of TIG (0.5/1/2 × MIC), MEM (0.5/1 × MIC), and IPM (0.5/1 × MIC) against the CRE isolates: (a–c) *bla*_IMP-4_-carrying CRECL42 strain; (d–f) *bla*_IMP-26_-carrying CRECL60 strain; (g–i) *bla*_IMP-4_-carrying CRKP294 strain; and (j–l) *bla*_IMP-4_-carrying CRECL352 strain.

To determine whether the combination of TIG/MEM or TIG/IPM has a dynamic synergistic effect against IMP-producing strains, we performed time-kill experiments with the results shown in Fig. 6. The time-kill results showed that MEM (0.5/1 × MIC) or IPM (0.5×/1 × MIC) alone had bactericidal activity against the CRECL352 strain, with no bacterial regeneration at 24 h (Fig. 6j through l). MEM (0.5 × MIC) or IPM (0.5 × MIC) alone had bactericidal activity against the CRECL60 and the CRKP294 strains at 4 h (Fig. 6d through i), with bacterial regeneration after 4 h. MEM (1× MIC) alone had bactericidal activity against the CRECL60 and the CRKP294 strains at 2–8 h (Fig. 6d through i), with bacterial regeneration after 8 h; IPM (1 × MIC, 4–12 h) alone also had bactericidal activity with CRECL60 strain regenerating at 12 h and CRKP294 strain regenerating at 4 h (Fig. 6d through i). however, IPM (1× MIC) alone had bactericidal activity against the CRECL42 strain at 4 h, with bacterial regeneration after 4 h (Fig. 6a through c) . For the time-kill assay of the combined agents, the results showed the bactericidal activity and synergistic effect of TIG (1× MIC)/MEM (1× MIC) against the CRECL42 strain at 12 h (Fig. 6b). Moreover, TIG (0.5/1/2× MIC)/IPM (1× MIC) or TIG (2× MIC)/IPM (0.5× MIC) showed bactericidal activity and synergistic effect against the CRECL60 strain (Fig. 6d through f).

## DISCUSSION

CRE strains, which exhibit a multidrug-resistant or extensively drug-resistant pattern, are considered a serious threat to public health, and there are few effective treatment options for the resulting infections. We isolated two IMP-4-producing CRECL strains and one IMP-4-producing *K. pneumoniae* from 259 clinical CRE strains, and it seems that IMP-4 is more prevalent in CRECL strains (9, 15); IMP-26 is less reported nationally and mostly found in *P. aeruginosa* (8, 16), but our study found its detection from CRECL (17, 18). The results of the conjugation assays showed that only the pIMP4-ECL42 plasmid could be successfully transferred into *E. coli* 600, probably because IncC is a self-transmissible plasmid and can mediate the co-transmission of *bla*_IMP-4_ and other ARGs (19–21), and IncC is also one of the most predominant plasmid replicon type carrying the *bla*_IMP-4_ (3, 22). However, the pIMP4-ECL352 (IncP1) plasmid has shown strong stability in this study because IncP1 is one of the most stable plasmid replicon types known to date (23). Although the pIMP4-ECL42 and the pIMP4-ECL352 plasmids had a similar genetic environment for *bla*_IMP-4_ (Fig. 2, bottom), the pIMP4-ECL352 plasmid was not successfully transferred into the recipient strain, so the IncC-type plasmid is more likely to mediate the transmission of *bla*_IMP-4_ by the following structure: *acc(6’)-lb-qacG2-bla*_IMP-4_-*intl1*. The IncU plasmid was extremely unstable according to plasmid stability, which may account for the failure of CRKP294 conjugation. Moreover, the IncHI2 type plasmid is a self-transmissible plasmid carrying multiple ARGs (it is also the main replicon type carrying *mcr-9*) (24, 25), but the pIMP26-ECL60 plasmid (carrying *bla*_IMP-26_ and *mcr-9*) was not successfully transferred into the recipient strain in our study, and further studies are needed to determine the possible reasons for this. Analysis of the T4SS first revealed that only the CRECL42 strain contained the *virD* T4SS (Fig. 3), so we speculate that the *virD* T4SS may play a role in plasmid transfer, which is consistent with the study by Álvarez-Rodríguez et al. (26). Second, compared to the CRECL42 strain, the relative expression of *traD* was significantly reduced in the CRECL60 and CRKP294 strains (Fig. 4); therefore, we suggested that the high expression of the *traD* may have facilitated the transfer of the pIMP4-ECL42 plasmid.

The pIMP26-ECL60 plasmid showed no significant plasmid loss after serial subculture for 5 days with or without the addition of the conjugate inhibitor linoleic acid (Fig. 5b), and it appears that reducing antibiotic use alone may not be sufficient to reverse resistance. However, the pIMP4-ECL42 plasmid showed a gradual increase in plasmid loss rate and a decrease in conjugation frequency upon the addition of linoleic acid (Fig. 5a; Fig. S2), suggesting that conjugation transfer may promote the stability of the IncC *bla*_IMP-4_-carrying plasmid. Therefore, combining inhibition of conjugation and promotion of plasmid loss would be an effective strategy to limit the persistence of the IncC *bla*_IMP-4_-carrying plasmid.

Our experimental results suggest that there may be no correlation between the *bla*_IMP-4_-carrying CRE strains and the intensity of biofilm formation, which is consistent with Arikawa et al., who reported that carbapenem resistance does not affect the amount of biofilm formation (27). Serum resistance test showed that *bla*_IMP-4_- and *bla*_IMP-26_-carrying strains were not associated with survival in complement-inactivated serum, which may indicate that there is no significant relationship between the degree of infection caused by CRE carrying *bla*_IMP-4_ and *bla*_IMP-26_ and whether the bacteria produce IMP-type carbapenemases. In summary, we have reason to believe that *bla*_IMP-4,26_ does not affect the virulence of CRE, and further studies should be conducted to make the conclusion more meaningful.

IMP is an IPM hydrolase. However, in our setting, the IMP-producing strains showed low MIC values for IPM (1–4 mg/L), which is consistent with the study by Thomson et al. (28). The results of the checkerboard assay showed that none of the TIG-based combinations exhibited synergistic effects. There was no significant bacteriostatic activity of TIG alone in the time-kill assay, which is consistent with the study by Tsala et al. (29). However, the combination of TIG/IPM showed bactericidal activity and synergistic effect against the *bla*_IMP-26_-carrying strain *in vitro*. In addition, IPM (1× MIC) alone showed bactericidal or bacteriostatic activity for all IMP-producing strains at 4–12 h. These findings suggest that pharmacodynamic interactions differ between static and dynamic concentrations *in vitro*. Previous studies found that carrying *bla*_IMP-4_ strains isolated from patients exhibited low levels of resistance to imipenem (MIC ≤4 mg/L) and then patients were given intravenous imipenem (20 mg/kg) every 12 h (10–14 days), and eventually the patients’ symptoms improved and they were discharged (30). Therefore, we suggest that the clinical use of antibiotics can be selected according to the MIC value of the strain. For CRE strains with low imipenem MIC values (MIC ≤4 mg/L), although they carry *bla*_IMP-4_, they can still use imipenem alone (short-acting), while the *bla*_IMP-26_-carrying strains can be treated with a combination of TIG and IPM (long-acting).

A minor limitation of our study is that we only studied IMP-producing strains among CRE strains isolated from two regions, which is probably the reason why the results we found for prevalence (1.54%) were lower than in previous studies (3.6%) (31). In conclusion, conjugation transfer of IncC *bla*_IMP-4_-carrying plasmid promotes plasmid stability; therefore, inhibition of conjugation transfer, as well as promotion of plasmid loss, may be potential ways to suppress the persistence of this plasmid. Although the IncP1 *bla*_IMP-4_-carrying plasmid could not be transferred by conjugation, it remained stable after successive passages and could be served as a reservoir of ARGs. Moreover, the IPM alone or TIG/IPM combination showed a good bactericidal effect against IMP-producing strains, which provides a potential therapeutic option for IMP-producing strains. Our results support further appropriate trials of antibiotics to determine the best treatment and suggest the importance of monitoring the prevalence of IMP-producing strains.

## MATERIALS AND METHODS

### Bacterial isolates, antimicrobial susceptibility testing, and clinical data collection

We included four IMP-producing non-duplicate CRE strains from 259 clinical CRE strains. These isolates were collected from two tertiary teaching hospitals, namely the First Affiliated Hospital of Jiamusi University (60 strains, April 2016 to August 2018) and Yongchuan Hospital of Chongqing Medical University (199 strains, June 2018 to December 2020), and the isolates were identified using a VITEK-2 Compact automatic microbiology analyzer (bioMérieux, France). The minimum inhibitory concentrations (MICs) of seven antibiotics were evaluated following the Clinical and Laboratory Standards Institute guidelines (M100-S30, 2020), and the results were interpreted according to these guidelines, except that colistin and tigecycline resistance was defined according to the European Committee on Antimicrobial Susceptibility Testing (version 10.0) criteria (https://eucast.org/clinical_breakpoints/). Strains that are resistant to meropenem, imipenem or ertapenem or produce carbapenemase are defined as CRE strains. Metadata including specimen type and patients’ clinical outcomes were recorded.

### PCR and sequencing

Total DNA was extracted from each strain using the boiling method and used as a template in PCR experiments (32). Resistance genes were detected by PCR, and all primers refer to previous studies (32, 33). The positive amplification products were sequenced using Sanger sequencing and the sequences obtained were compared with those available on the Internet using the Basic Local Alignment Search Tool (BLAST) tool (http://www.ncbi.nlm.nih.gov/blast/).

### Nucleic acid extraction, WGS, and bioinformatic analysis

High-quality DNA was extracted using Qiagen kits. DNA was sequenced using the PromethION sequencing platform from Oxford Nanopore Technologies. *In silico* MLST was performed using PubMLST (https://pubmlst.org/). The plasmid replicon type was identified using Plasmidfinder 2.1 (https://cge.food.dtu.dk/services/PlasmidFinder/). Acquired ARGs were identified using ResFinder 4.1 (https://cge.food.dtu.dk/services/ResFinder/). The virulence-associated genes were identified using VirulenceFinder 2.0 (https://cge.food.dtu.dk/services/VirulenceFinder/). T4SS was identified using oriTfinder (34). Comparative analysis of plasmids carrying *bla*_IMP_ was determined using the BLAST Ring Image Generator (BRIG). Sequence alignments were performed using BLAST and visualized using Easyfig v 2.2.5.

### Conjugation assays

Conjugation assay was performed using the membrane bonding method as described previously (32). Briefly, CRE strains carrying the *bla*_IMP_ were used as donors and *E. coli* 600 (rifampicin-resistant) was used as the recipient strain. Transconjugants were selected on Mueller–Hinton (MH) agar supplemented with rifampicin (1,200 mg/L) and meropenem (1 mg/L). The presence of ARGs in transconjugants was confirmed using PCR and Sanger sequencing.

### mRNA extraction and RT-qPCR

Total RNA was extracted from overnight cultures using the PureLink™ RNA Mini Kit (Thermo Fisher Scientific) according to the manufacturer’s instructions. The relative expression level of *traD* was determined by quantitative real-time PCR (RT-qPCR) using TB Green premix Ex Taq (TaKaRa, Kyoto, Japan) on the CFX96 RealTime PCR system. The lists of primers designed by using Primer are listed in Table S1. Relative gene expression levels were calculated using the 2 ^-ΔΔCT^ formula with the *rpoB* gene as the internal control. The experiment was repeated on three separate occasions.

### Plasmid stability assays

Plasmid stability assay was determined as previously described with slight modifications (35). Briefly, four IMP-producing strains and one transconjugant (J42) were cultured overnight in Luria-Bertani (LB) broth. Subsequently, cultures were diluted 1:20 into fresh antibiotic-free LB broth and passaged every 12 h until at least 50 generations. Meanwhile, approximately 100 colonies were randomly selected every five generations and inoculated on MH agar plates with or without meropenem.

To investigate the effect of conjugating transfer on the stability of plasmids carrying *bla*_IMP_, strains were passaged in LB broth supplemented with final concentrations of 2.5 and 5 mM of the conjugate inhibitor linoleic acid for 5 consecutive days (36).

### Plasmid elimination

Plasmid elimination was performed as previously described with slight modifications (37). Briefly, overnight cultures of IMP-producing CRE strains were inoculated into 2 mL LB with shaking at 45°C for 18 h and then inoculated into LB broth containing 1% SDS with shaking at 37°C for 18 h. After four cycles of this, 100 colonies were randomly selected and inoculated onto MH agar plates with or without meropenem.

### Biofilm formation and serum complement-mediated killing assays

The biofilm-forming ability of bacteria was analyzed using 96-well plates as previously described (38). Briefly, 10 µL of bacterial suspension and 190 µL of LB broth were inoculated into 96-well plates and incubated at 37°C for 24 h. Five replicates of each strain were prepared. LB broth was removed from the wells and 0.1% crystal violet was added to stain the bound bacteria. The wells were washed with phosphate-buffered saline (PBS) and the dried biofilm was solubilized in 200 µL of ethanol. The absorbance was measured at 570 nm using a microplate reader.

The serum complement-mediated killing assay was performed as previously described (38). Briefly, fresh serum from 10 healthy volunteers was collected and mixed and stored at −80°C (informed consent was obtained before collection). Approximately 10^6^ colony-forming units (CFU)/mL of bacterial suspension was mixed with serum at a volume ratio of 1:3 and incubated at 37°C for 3 h. The cultured bacterial suspensions were plated on MH agar plates each hour and colonies on the plates were counted after 24 h of incubation at 37°C. Each strain was tested three times.

### Checkerboard technique

The combination effect of different antimicrobial combinations was evaluated by the microdilution broth checkerboard method (39). The following combinations were tested: PB-TIG/MEM/ IPM/AMK/LEV, TIG-MEM/IPM/AMK/LEV, MEM-AMK/LEV, and AMK-LEV. The concentration ranges were based on the MICs determined above. 50 µL of each antibiotic at five increasing (4-fold) concentrations (0.125 to 2 × MIC) were used, and each well was inoculated with 100 µL bacteria suspension (around 7.5 × 10^5^ CFU/mL) in a final volume of 200 µL with two biological replicates. The absorbance of bacterial culture at 570 nm was measured using a microplate reader. The effects of the antimicrobial combinations were defined according to the fractional inhibitory concentration index (FICI) (40).

### Time-kill assays

The bactericidal activities of antibiotics against IMP-producing CRE strains were determined by the time-kill method (41). The initial bacterial inoculum contained 5 × 10^5^ CFU/mL in fresh MH broth. The following antibiotic concentrations were used: TIG (0.5/1/2× MIC) and MEM/IPM (0.5/1× MIC). Cultures were incubated for 0, 2, 4, 8, 12, and 24 h and were diluted and plated on MH agar plates. A reduction in colony count ≥3 log10 compared to the initial CFU/mL was considered a bactericidal effect, while a reduction of <3 log10 CFU/mL compared to the initial CFU/mL was defined as a bacteriostatic effect (41). A reduction in CFU/mL of ≥2 log10 for the co-cultures compared to the most active single agent broth at the same time point was defined as a synergistic effect, while an increase of >2 log10 for the co-cultures was interpreted as an antagonistic effect (41).

## Data Availability

WGS data for CRECL42, CRECL60, CRKP294, and CRECL352 have been deposited in GenBank under accession no. CP082147.1-CP082152.1, CP083234.1-CP083236.1, CP083445.1-CP083447.1, and CP083709.1-CP083714.1, respectively.

## SUPPLEMENTAL MATERIAL

Figure S1 spectrum.02318-23-s0001.tif Phylogenetic analysis of plasmids harbouring *bla*_IMP_.

Figure S2 spectrum.02318-23-s0002.tif Conjugation experiments on the CRECL42 strain in the presence of 2.5 mM or 5 mM linoleic acid.

Figure S3 spectrum.02318-23-s0003.tif Results of biofilm formation.

Figure S4 spectrum.02318-23-s0004.tif Results of serum resistance assay.

Supplemental legends spectrum.02318-23-s0005.doc Legends for Figure S1 to Figure S4.

Table S1 spectrum.02318-23-s0006.doc Oligonucleotide sequences used in this work.

Table S2 spectrum.02318-23-s0007.doc Characterization of IMP-producing strains identified in this study.

Table S3 spectrum.02318-23-s0008.doc Distribution of resistance genes in 4 *bla*_IMP-4_- or *bla*_IMP-26_-carrying plasmids.

Table S4 spectrum.02318-23-s0009.doc Stability characteristics of IMP-producing strains identified in this study.

ASM does not own the copyrights to Supplemental Material that may be linked to, or accessed through, an article. The authors have granted ASM a non-exclusive, world-wide license to publish the Supplemental Material files. Please contact the corresponding author directly for reuse.
