# Supplementary figures and images for: Mechanism for transmission and pathogenesis of carbapenem-resistant Enterobacterales harboring the carbapenemase IMP and clinical countermeasures
Source: Microbiol Spectr. 2024 Jan 10;12(2):e02318-23. doi: 10.1128/spectrum.02318-23 (PMC10846200; doi:10.1128/spectrum.02318-23)

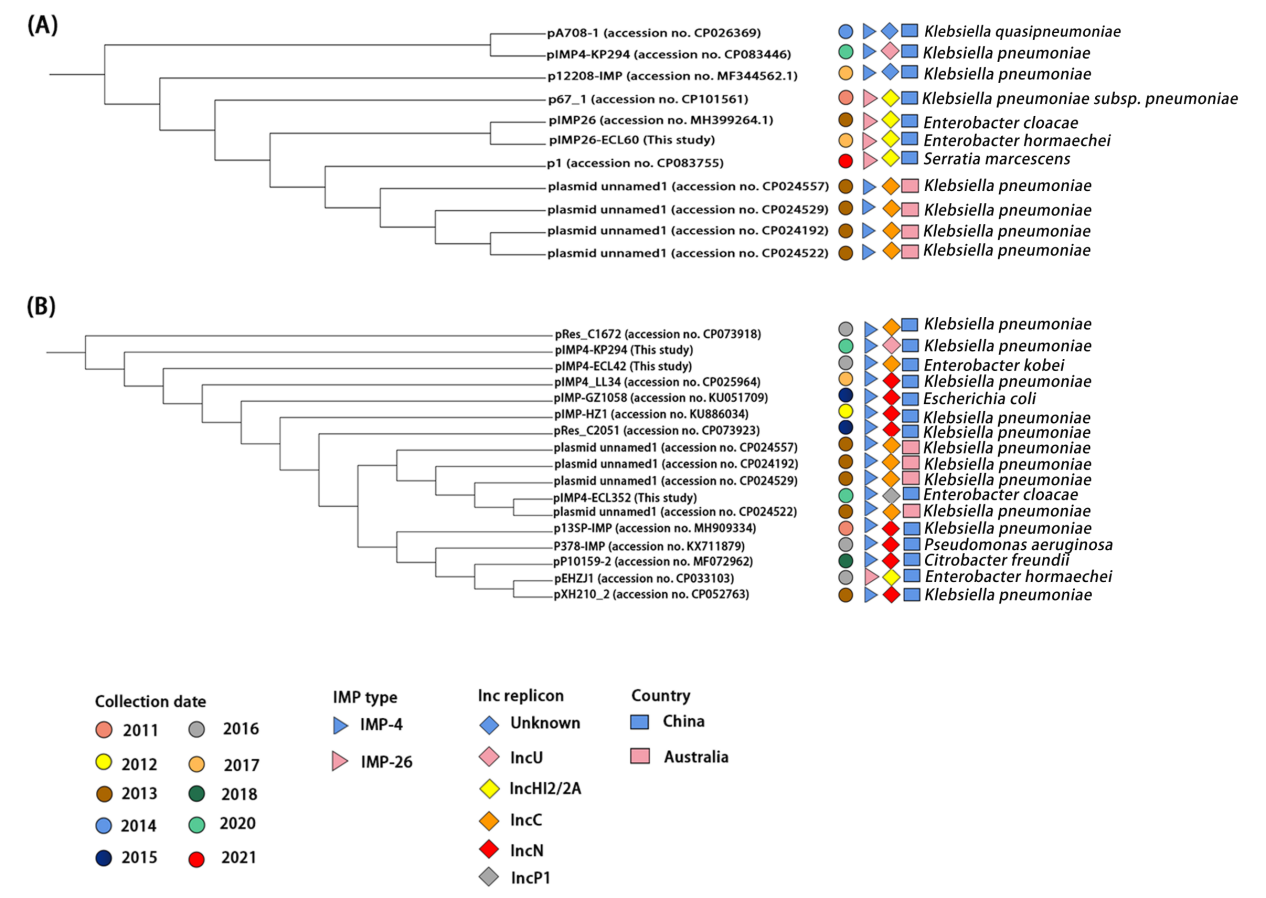

Supplement: Figure S1 — Phylogenetic analysis of plasmids harbouring blaIMP. [file spectrum.02318-23-s0001.tif]

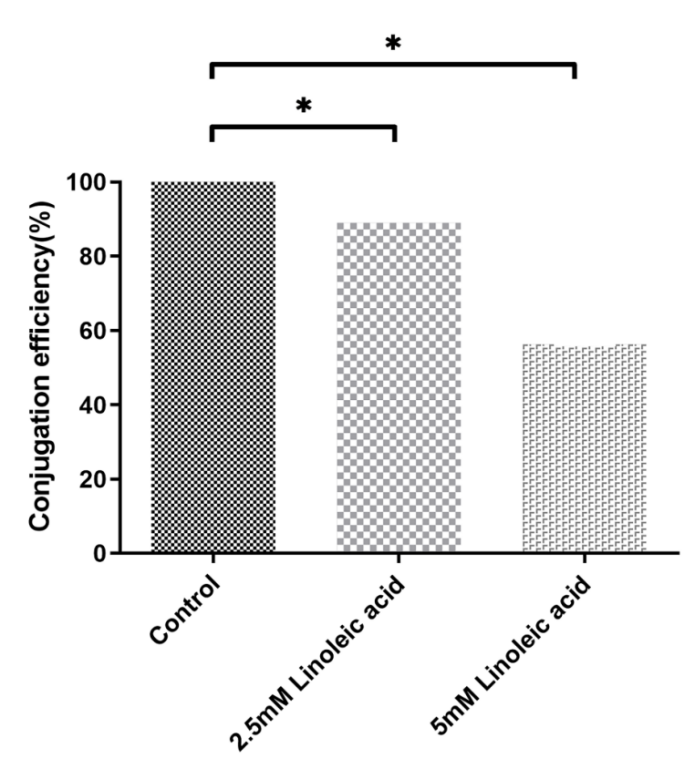

Supplement: Figure S2 — Conjugation experiments on the CRECL42 strain in the presence of 2.5 mM or 5 mM linoleic acid. [file spectrum.02318-23-s0002.tif]

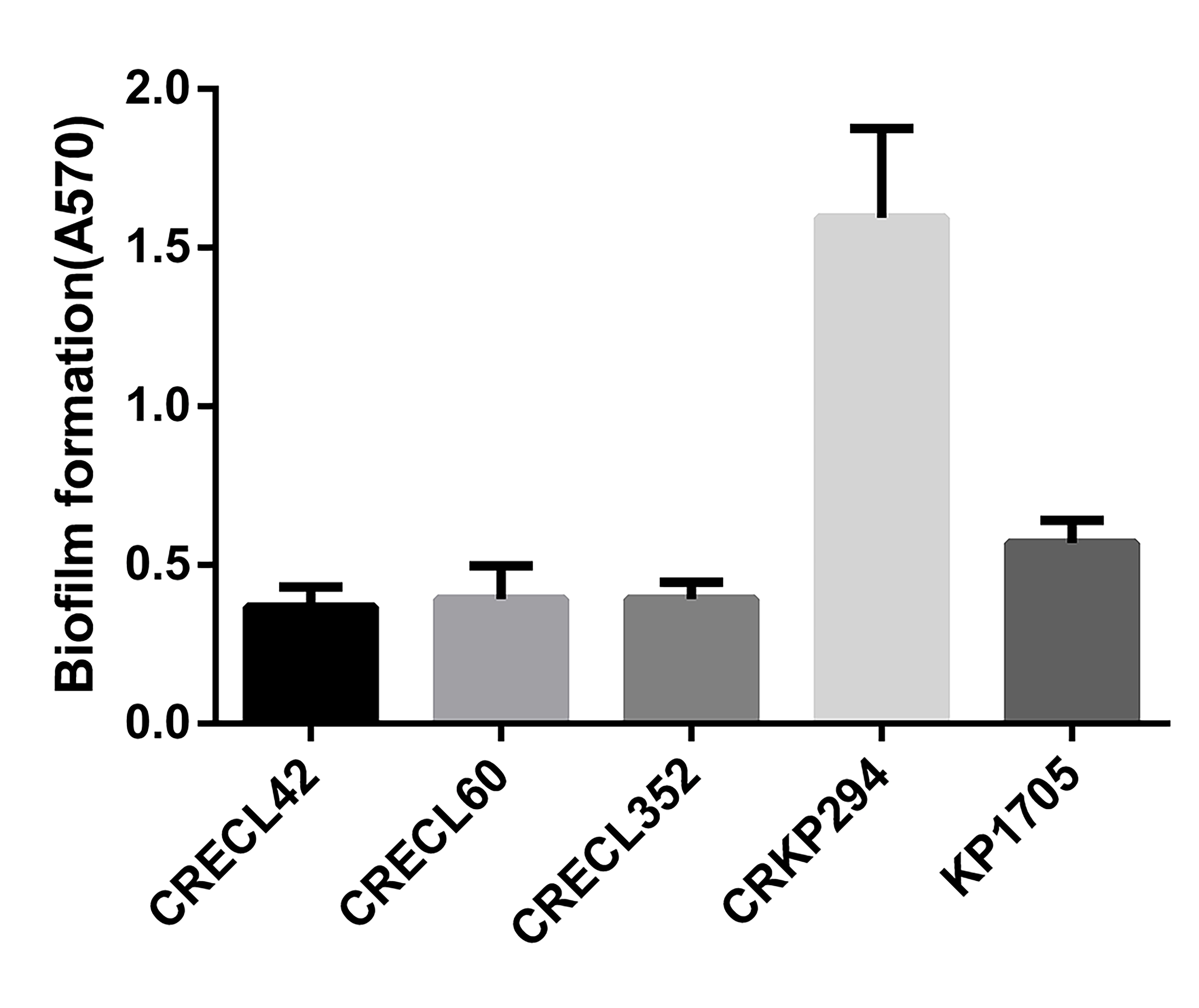

Supplement: Figure S3 — Results of biofilm formation. [file spectrum.02318-23-s0003.tif]
