# Supplementary material for: Mechanism for transmission and pathogenesis of carbapenem-resistant Enterobacterales harboring the carbapenemase IMP and clinical countermeasures
Source: Microbiol Spectr. 2024 Jan 10;12(2):e02318-23. doi: 10.1128/spectrum.02318-23 (PMC10846200; doi:10.1128/spectrum.02318-23)
Supplement: Supplemental legends — Legends for Figure S1 to Figure S4. [file spectrum.02318-23-s0005.doc]

**Figure S1** Phylogenetic analysis of plasmids harbouring *bla*_IMP_. Plasmids harbouring *bla*_IMP_ from published and our institution isolates were clustered on the basis of sequences similarity.

**Figure S2** Conjugation experiments on the CRECL42 strain in the presence of 2.5 mM or 5 mM linoleic acid. “Conjugation efficiency” refers to the relative conjugation frequencies of the IncC *bla*_IMP-4_-carrying plasmid after adding the conjugation inhibitor linoleic acid. ^＊^, P≤0.05.

**Figure S3** Results of biofilm formation. Quantification of biofilm formation by measuring crystal violet uptake.

**Figure S4** Results of serum resistance assay. KP99 is *K. pneumoniae* strain (non IMP-producing) and is sensitive to antibiotics other than ampicillin.
