# Supplementary material for: Mechanism for transmission and pathogenesis of carbapenem-resistant Enterobacterales harboring the carbapenemase IMP and clinical countermeasures
Source: Microbiol Spectr. 2024 Jan 10;12(2):e02318-23. doi: 10.1128/spectrum.02318-23 (PMC10846200; doi:10.1128/spectrum.02318-23)
Supplement: Table S1 — Oligonucleotide sequences used in this work. [file spectrum.02318-23-s0006.doc]

**Table S1**

Oligonucleotide sequences used in this work

| gene | Primer sequence | length（bp） |
| --- | --- | --- |
| *traD*-CRKP294 | F: GGACCAGCAAGAACGGAATCAGTG | 80 |
|  | R: CCATAACCTCCAGCAAGCCATAGC |  |
| *traD*-CRECL60 | F: CAAGATGGCAGGAAGGTTGTAGGC | 85 |
|  | R: GCATTTCCGACAGAGTTGTGTTTGG |  |
| *traD*-CRECL42 | F: CGGACGATATGTGGCTTGGTAGTG | 116 |
|  | R: TTTGACCGTGGACTCTCTCCCTAC |  |
| *rpoB*-KP | F: CATCGGTCTGATTAACTC | 189 |
|  | R: GTTGGTCACTTTACGATA |  |
| *rpoB*-ECL | F: AAGGCGAATCCAGCTTGTTCAGC | 148 |
|  | R: TGACGTTGCATGTTCGCACCCATCA |  |
