# Supplementary material for: Mechanism for transmission and pathogenesis of carbapenem-resistant Enterobacterales harboring the carbapenemase IMP and clinical countermeasures
Source: Microbiol Spectr. 2024 Jan 10;12(2):e02318-23. doi: 10.1128/spectrum.02318-23 (PMC10846200; doi:10.1128/spectrum.02318-23)
Supplement: Table S2 — Characterization of IMP-producing strains identified in this study. [file spectrum.02318-23-s0007.doc]

**Table S2**

Characterization of IMP-producing strains identified in this study

| Strain name | | CRECL42 | CRECL60 | CRKP294 | CRECL352 | J42^a^ |
| --- | --- | --- | --- | --- | --- | --- |
| Clinical features | Region | Jiamusi | Jiamusi | Chongqing | Chongqing | - |
|  | Age/  sex | 59 yr/  female | 46 yr/  female | 60 yr/  female | 30 days/  male | - |
|  | Specimen | Blood | Blood | Secretions | Sputum | - |
|  | Diagnosis/  ward | Decompensated cirrhosis/EICU | Cerebral hemorrhage and pneumonia/  emergency ward | Wound infection/  Orthopedics (Trauma) | Premature baby, neonatal respiratory failure, and neonatal sepsis/NICU | - |
|  | Outcome | Death | Improvement | Discharge | Discharge | - |
| MIC^b^ | PB | 4 | 2 | 4 | 512 | 1 |
|  | TIG | 1 | 1 | 16 | 1 | ≤1/8 |
|  | MEM | 4 | 4 | 4 | 8 | 2 |
|  | AMK | ≥2048 | 16 | 4 | 2 | 2048 |
|  | LEV | 1/8 | 1/2 | 4 | ≤1/32 | ≤1/8 |
|  | IPM | 2 | 1 | 1 | 4 | 1 |
|  | CAZ/AVI | 256,4 | ≥256,4 | 128,4 | 32,4 | 128,4 |
| FICI | PB+TIG | 1.5 | 1.03125 | 0.625 | - | - |
|  | PB+MEM | 0.625 | 1 | 0.5625 | - | - |
|  | PB+IPM | 0.625 | 0.5 | 0.75 | - | - |
|  | PB+AMK | - | 0.75 | 0.625 | - | - |
|  | PB+LEV | 1 | 1.125 | 0.5625 | - | - |
|  | TIG+MEM | 0.625 | 1 | 1.125 | 1.03125 | - |
|  | TIG+IPM | 0.75 | 0.53125 | 1 | 1 | - |
|  | TIG+AMK | - | 1 | 1 | 1.5 | - |
|  | TIG+LEV | 0.53125 | 1 | 1.0625 | 1.0625 | - |
|  | MEM+AMK | - | 0.5 | 1 | 0.75 | - |
|  | MEM+LEV | 0.25 | 0.625 | 0.75 | 1.25 | - |
|  | AMK+LEV | - | 0.5 | 1 | 0.625 | - |

a, *E. coli* transconjugant strain; “-”, None; EICU, emergency intensive care unit; NICU, neonatal intensive care unit; b, mg/L; PB, polymyxin B; TIG, tigecycline; MEM, Meropenem; AMK, Amikacin; LEV, levofloxacin; IPM, Imipenem; CAZ/AVI, ceftazidime/avibactam. FICI, fractional inhibitory concentration index. The PB combination approach for the CRECL352 strain and the AMK combination approach for the CRECL42 strain were not used because they were highly resistant to PB and AMK, respectively.
