# Supplementary material for: Mechanism for transmission and pathogenesis of carbapenem-resistant Enterobacterales harboring the carbapenemase IMP and clinical countermeasures
Source: Microbiol Spectr. 2024 Jan 10;12(2):e02318-23. doi: 10.1128/spectrum.02318-23 (PMC10846200; doi:10.1128/spectrum.02318-23)
Supplement: Table S3 — Distribution of resistance genes in 4 blaIMP-4- or blaIMP-26-carrying plasmids. [file spectrum.02318-23-s0008.doc]

**Table S3**

Distribution of resistance genes in 4 *bla*_IMP-4_-or *bla*_IMP-26_-carrying plasmids

| Plasmid name | | | pIMP4-ECL42 | pIMP26-ECL60 | pIMP4-KP294 | pIMP4-ECL352 |
| --- | --- | --- | --- | --- | --- | --- |
| Host strains | | | CRECL42 | CRECL60 | CRKP294 | CRECL352 |
| Size (bp) | | | 217,402 | 320,374 | 349,403 | 70,058 |
| Replicons | | IncC |  |  |  |  |
|  |  | IncHI2 |  |  |  |  |
|  |  | IncU |  |  |  |  |
|  |  | IncP1 |  |  |  |  |
| Resistance  genes | β-lactams | *bla*_IMP-4_ |  |  |  |  |
|  |  | *bla*_IMP-26_ |  |  |  |  |
|  |  | *bla*_SFO-1_ |  |  |  |  |
|  |  | *bla*_OXA-1_ |  |  |  |  |
|  |  | *bla*_DHA-1_ |  |  |  |  |
|  |  | *bla*_SHV-12_ |  |  |  |  |
|  |  | *bla*_TEM-1B_ |  |  |  |  |
|  |  | *bla*_PER-1_ |  |  |  |  |
|  | Aminoglycosides | *aac(6')-lb3* |  |  |  |  |
|  |  | *armAC* |  |  |  |  |
|  |  | *aph(3'')-lb* |  |  |  |  |
|  |  | *aac(6')-llc* |  |  |  |  |
|  |  | *aph(6)-ld* |  |  |  |  |
|  |  | *aadA5* |  |  |  |  |
|  |  | *aac(3)-IId* |  |  |  |  |
|  | Trimethoprims | *dfrA19* |  |  |  |  |
|  |  | *dfrA1* |  |  |  |  |
|  | Sulfonamides | *sul1* |  |  |  |  |
|  | Tetracyclines | *tet(D)* |  |  |  |  |
|  |  | *tet(A)* |  |  |  |  |
|  | Quinolones | *aac(6’)-Ib-cr* |  |  |  |  |
|  |  | *qnrS1* |  |  |  |  |
|  |  | *qnrB4* |  |  |  |  |
|  | Macrolides | *msr(E)* |  |  |  |  |
|  |  | *mph(E)* |  |  |  |  |
|  |  | *ere(A)* |  |  |  |  |
|  |  | *mph(A)* |  |  |  |  |
|  | Colistin | *mcr-9* |  |  |  |  |
|  | Rifampicin | *ARR-3* |  |  |  |  |

^a^ Rectangles marked in red indicate the presence of the corresponding genes listed on the left.
