# Supplementary material for: Mechanism for transmission and pathogenesis of carbapenem-resistant Enterobacterales harboring the carbapenemase IMP and clinical countermeasures
Source: Microbiol Spectr. 2024 Jan 10;12(2):e02318-23. doi: 10.1128/spectrum.02318-23 (PMC10846200; doi:10.1128/spectrum.02318-23)
Supplement: Table S4 — Stability characteristics of IMP-producing strains identified in this study. [file spectrum.02318-23-s0009.doc]

**Table S4**

Stability characteristics of IMP-producing strains identified in this study

| Strains | Plasmid retention rate (%)^a^ | Plasmid elimination rate (%)^b^ |
| --- | --- | --- |
| CRECL42 | 15 | 11.7 |
| CRECL60 | 8.8 | 10 |
| CRKP294 | 0^c^ | 100 |
| CRECL352 | 100 | 0 |
| J42 | 85 | 0 |

^a^The plasmid retention rate was calculated by comparing the number of colonies on MH agar plates containing meropenem with the number of colonies on antibiotic-free MH agar; ^b^The plasmid elimination rate was determined as the difference between the number of colonies on the antibiotic-free plate and the plate containing meropenem divided by the number of colonies on the antibiotic-free plate; ^c^All plasmids were lost after 30 generations of natural passages.
